# Effects of highly active antiretroviral therapy with nelfinavir in vertically HIV-1 infected children: 3 years of follow-up. Long-term response to nelfinavir in children

**DOI:** 10.1186/1471-2334-6-107

**Published:** 2006-07-11

**Authors:** Salvador Resino, Beatriz Larrú, Jose Ma Bellón, Rosa Resino, Ma Isabel de José, Marisa Navarro, Juan Antonio Léon, José Tomás Ramos, Ma José Mellado, Ma Ángeles Muñoz-Fernández

**Affiliations:** 1 Laboratorio de Inmuno-Biología Molecular, Hospital General Universitario "Gregorio Marañón", Madrid, Spain; 2 Pediatría-Infecciosas, Hospital Universitario "La Paz", Madrid, Spain; 3 Pediatría-Infecciosas, Hospital General Universitario "Gregorio Marañón", Madrid, Spain; 4 Pediatría-Infecciosas, Hospital Universitario "Virgen de Rocío", Sevilla, Spain; 5 Inmuno-Pediatría, Hospital Universitario "12 de Octubre", Madrid, Spain; 6 Pediatría-Infecciosas, Hospital Universitario "Carlos III", Madrid, Spain

## Abstract

**Background:**

Antiretroviral treatment (ART) in children has special features and consequently, results obtained from clinical trials with antiretroviral drugs in adults may not be representative of children. Nelfinavir (NFV) is an HIV-1 Protease Inhibitor (PI) which has become as one of the first choices of PI for ART in children. We studied during a 3-year follow-up period the effects of highly active antiretroviral therapy with nelfinavir in vertically HIV-1 infected children.

**Methods:**

Forty-two vertically HIV-infected children on HAART with NFV were involved in a multicentre prospective study. The children were monitored at least every 3 months with physical examinations, and blood sample collection to measure viral load (VL) and CD4+ cell count. We performed a logistic regression analysis to determinate the odds ratio of baseline characteristics on therapeutic failure.

**Results:**

Very important increase in CD4+ was observed and VL decreased quickly and it remained low during the follow-up study. Children with CD4+ <25% at baseline achieved CD4+ >25% at 9 months of follow-up. HIV-infected children who achieved undetectable viral load (uVL) were less than 40% in each visit during follow-up. Nevertheless, HIV-infected children with VL >5000 copies/ml were less than 50% during the follow-up study. Only baseline VL was an important factor to predict VL control during follow-up. Virological failure at defined end-point was confirmed in 30/42 patients. Along the whole of follow-up, 16/42 children stopped HAART with NFV. Baseline characteristics were not associated with therapeutic change.

**Conclusion:**

NFV is a safe drug with a good profile and able to achieve an adequate response in children.

## Background

The introduction of highly active antiretroviral therapy (HAART) has lead to a significant decrease in mortality and disease progression in HIV-1 infected children and adolescents [1,2]. Antirretroviral treatment (ART) in children has special features and consequently, results obtained from clinical trials in adults may not be representative of children and more studies about ART effectiveness in the paediatric age are needed.

Nelfinavir (NFV) is an HIV-1 Protease Inhibitor (PI). When combined with other antiretroviral drugs, it has been associated with immunologic and virologic responses in antiretroviral therapy-naïve and experienced adults and children with a good tolerance profile [3,4]. The challenge to give it weigh twice daily instead of the previous schedule of three times per day [5,6] and the weaker relationship between NFV and changes on metabolism, have become NFV as a good choice for ART in children [7]. However further analysis, like our study, to assess the association between baseline characteristics and virological or therapeutical failures are needed.

## Methods

### Population and study design

This is a multicentre prospective study on a cohort of 42 vertically HIV-infected children on HAART with NFV recruited between May 1997 to October 2001, and followed-up until October 2004 in 5 Spanish hospitals. This study was conducted according to the declaration of Helsinki and approved by the Ethical Committees of all hospitals involved.

The inclusion criteria were: a) VL >1,000 copies/mL at baseline, b) at least 6 months of follow-up, c) starting HAART for the first time with nelfinavir (25–35 mg/Kg, three times per day), d) having received mono or dual nucleoside therapy before starting HAART. e) No CD4+ cell count and age restrictions. All vertically HIV infected children in Madrid are enrolled in the same cohort, which has 276 patients. We selected those patients who started HAART with NFV, 72 out of 276. Nevertheless, from these 72 infants, 42 had previous treatment and 14 were naïve. We selected only the 42 experienced children in this study.

The children were monitored under a standardized form at least every 3 months with physical examinations, and serial measurements of CD4+ T-cells and VL as described below [8]. There was not a uniform approach regarding antiretroviral treatment in the background regimen given together with NFV. During the follow-up we recorded all the clinical events and side effects related to the use of NFV.

VL was measured in plasma using the Amplicor Monitor assay. (Amplicor monitor, Roche Diagnostic Systems, Brandenburg, NJ, USA). The limit of quantification (LOQ) was 400 copies/mL. Adherence was measured by each clinician by pill count methods and interviews with parents or tutors.

### Statistical analysis

Analysis of data was performed by on-treatment analysis of observed data. Data from patients who were lost to follow-up were censored during the last visit. Endpoint for therapeutical failure was defined as any of the following: a) failure to reduce VL < 1 log_10 _during the first 12 weeks of HAART, unless it was < lower LOQ; and b) failure to achieve a VL < LOQ after 24 weeks of HAART. c) change of NRTI or additional NNRTI or PI in HAART regimen during the first 24 months of follow-up, while taking NFV; d) Stop of HAART with NFV during the first 24 months of follow-up. We performed a multivariate logistic regression analysis to determinate the odds ratio (OR) of baseline characteristics (%CD4^+^, VL, and age at baseline, previous ART protocol, and new drugs in HAART regimen) on therapeutical failure.

Moreover, we also consider as therapeutic failure during the whole follow-up: a) achieve a decrease of 1 log_10 _VL, unless it was < lower LOQ; b) achieve VL < LOQ. c) change of NRTI or additional NNRTI or PI in HAART; d) stop of HAART with NFV. e) rebound of two consecutive VL ≥ LOQ after achieving a VL < LOQ. This treatment failure was analyzed by Kaplan-Meier method and multivariate Cox regression analyses to assess the hazard ratio (HR) values of baseline characteristics.

All tests were two-tailed with P-values < 0.05 considered significant. Statistical analysis was performed by SPSS 12.0 software (SPSS INC, Chicago, IL, USA).

## Results

### Characteristics of HIV-1-infected children

During the study period (median: 41 (min: 6; max: 71.3) months), a total of 42 children with previous ART started HAART with NFV (Table 1). More than 50% of those children had diagnosis of AIDS previous to baseline; 29/42 of children had CD4^+ ^T-cells <25% whereas 14/42 had VL >50.000 copies/ml.

**Table 1:** Characteristics of clinical, immunological, and virological parameters, and antiretroviral treatment of vertically HIV-1-infected children.

	**Previous-ART**
**N. of HIV-children**	42
**Age (years)**^(a)^	6.7 ± 0.64 (0.5; 16.1)
**Male**^(b)^	16 (38.1%)
**AIDS diagnosis (CDC)**^(b)^	23 (54.7%)
**Baseline CD4**^+^**cell count**	
% CD4^+(a)^	23.2 ± 1.8 (0.8; 49.7)
15 – 25% CD4^+(b)^	13 (22%)
<15 % CD4^+(b)^	16 (27.1%)
**Baseline HIV-RNA level**	
log_10 _VL (copies/mL)^(a)^	4.52 ± 0.11 (3.22; 6.84)
VL >50,000 copies/mL^(b)^	14 (33.3%)
**ART with NRTI prior-HAART**^(b)^	
Monotherapy	39 (92.9%)
Therapy combined	3 (7.1%)
**HAART regimen at baseline**^(b)^	
3TC + d4T + NFV	27 (64.3%)
AZT + 3TC + NFV	6 (14.3%)
d4T + ddI + NFV	9 (21.49%)
New NRTI on HAART	
0 NRTI	14 (33.3%)
1 NRTI	14 (33.3%)
2 NRTI	14 (33.3%)
**HAART regimen during follow-up**^(b)^	
Global adherence (>90%)	100%
Change of drugs on first line of HAART	9 (21.4%)
Change of NRTI	2 (4.8%)
Adding a NNRTI	4 (9.5%)
Adding a PI	3 (7.1%)
Stop of HAART with NFV	16 (38.1%)
Change of NFV by other PI	12 (28.6%)
Interruption of HAART	4 (9.5%)

Values are expressed as: a) mean ± s.e.m. (min; max), and b) absolute (percentage). VL: viral load; CDC: Center for Disease Control. HAART: highly active antiretroviral therapy.

The HAART protocol used was 2 NRTI plus NFV and 28/42 of children changed NRTI in first line of HAART, because all of them have taken previous NRTI regimens.

### Virological and immunological response

**Figure 1:**
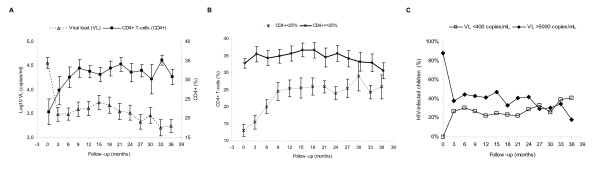
Summary of viral load (VL) and %CD4^+ ^evolution during follow-up. **A**: mean of log_10 _VL (copies/ml) and CD4^+^ T-cells percentage. **B**: mean of CD4^+^ T-cells percentage according to CD4^+^ at baseline (< or > 25%). **C**: percentage of HIV-infected children with VL ≤400 copies/ml and VL >5000 copies/ml.

A very important increase in CD4+ was observed and VL decreased quickly and sustained (Figure 1A). Children with CD4+ <25% at baseline had a CD4+ recovery and they achieved CD4+ >25% 9 months of follow-up, but these children did not achieve similar values of CD4+ than children with CD4+ ≥25% at baseline (Figure 1B). However, we did not find differences in VL control between them (data not shown). Moreover, HIV-infected children who achieved uVL (≤400 copies/ml) were less than 40% during follow-up (Figure 1C). Nevertheless, HIV-infected children with VL >5000 copies/ml were less than 50% during the follow-up study (Figure 1C).

**Figure 2:**
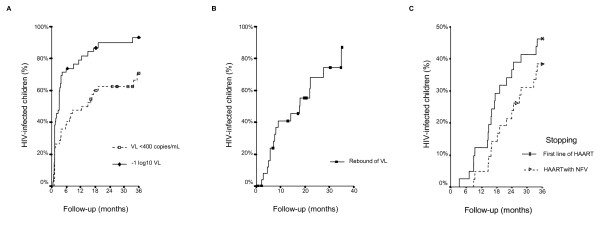
Summary of Kaplan-Meier curves of HIV-infected children on HAART during follow-up. **A**: to achieve VL <400 copies/ml for the first time or to achieve a decrease of -1 log_10 _VL. **B**: rebound of viral load after achieving VL ≤400 copies/ml. **C**: to change NRTI or additional NNRTI or PI in HAART regimen in the first line of HAART; or stop HAART with nelfinavir.

In this follow-up study, 38/42 children achieved a decrease in VL < 1 log_10 _and 28/42 children achieved uVL anytime during follow-up (Figure 2A). The median time to achieve a decrease in -1 log_10 _VL was 3 ± 0.64 months, and to achieve uVL was 12.4 ± 4.87 months.

Only baseline VL and previous ART protocol [Combined therapy (CT) or Mono-therapy (MT)] were important factors to predict VL control during follow-up. The HR of baseline VL to achieve uVL was 0.33 (CI95%: 0.16; 0.68; p = 0.003) per log_10 _VL at baseline and HR of previous ART protocol (CT vs. MT) to achieve uVL was 0.18 (CI95%: 0.05; 0.69; p = 0.013). HIV-infected children with VL >50,000 copies/mL at baseline had a negative likelihood of 3.34 (CI95%: 1.15; 9.67; p = 0.014) to achieve uVL. After achieving uVL, 17/28 children had a rebound of VL >400 copies/ml with a mean time of 17.7 ± 6.4 months (Figure 2B).

Moreover, 19/42 children changed of NRTI or additional NNRTI or PI in HAART regimen in the first line of HAART, with a mean of time of 30.4 ± 1.32 months. Besides, 16/42 children stopped HAART with NFV with a mean of time of 27.4 ± 1.7 months by Kaplan-Meier method (Figure 2C). Baseline characteristics were not associated with therapeutic change.

### Endpoint of therapeutical failure

Virological failure at defined end-point was confirmed in 30/42 patients. Besides, 20/42 children had virological failure defined as a reduction in VL <1 log_10 _during the first 12 weeks of HAART; and 25/42 children had virological failure to achieve a VL < LOQ after 24 weeks of HAART. When these 3 criteria of virological failure were used combined, we only found that baseline VL had a value of OR of 3.76 (CI95%: 1.13; 12.47; p = 0.03) per log_10 _and VL >50,000 copies/mL at baseline had a value of OR of 8.57 (CI95%: 1.31; 56.3; p = 0.025) to achieve a VL < LOQ after 24 weeks of HAART.

On HAART with NFV, 9/42 children changed of NRTI or additional NNRTI or PI in HAART regimen during the first 24 months of follow-up and 15/42 children stopped HAART with NFV during the first 24 months of follow-up.

### Clinical events

Among all patients, one child died and two children developed AIDS during the follow-up. Only four patients experienced side effects; diarrhoea in two patients, rash and hypertransaminasemia in one infant and hypercholesterolemia in other child.

The adherence of the antiretroviral drugs was measured by each paediatrician by examination of the dose taken by each child and through interviews with their parents or tutors. Overall, the adherence was >90% among all the infants included in our study during the follow-up. Besides, HAART with NFV was well tolerated.

## Discussion

Our study reveals that NFV is a safe drug with a good profile and active to achieve an adequate response in HIV-1 infected children. NFV, like other PI, is highly active in vitro, therefore its activity in vivo is limited by their rapid clearance [9]. Pharmacologic enhancement of PI with low doses of ritonavir (RTV) has been shown to increase drug concentrations [10]. The combination of lopinavir (LPV) and RTV, has been widely used on paediatric patients and it has demonstrated a great activity, even higher as those reported with NFV [11]. Nevertheless, LPV/RTV was not approved for administration to paediatric patients when our study was started [12]. Moreover, the lipid abnormalities and insulin resistance reported in adults with LPV/RTV with an unknown effect on children [13,14], the availability of a palatable form of NFV and the report of higher cross-resistance when LPV/RTV is used in a first line of HAART, has lead to an extensive use of NFV in paediatric age [15].

We recruited 42 infants who were not naïve to antiretroviral drugs. Nevertheless, NFV showed high efficacy in increasing the CD4+ cell count and early decreasing of VL among the three years of follow-up. Our results are similar to other studies which evaluate NFV efficacy among children [4,16].

The presence of higher virologic failures at the end of our study, 71.4% compared with data published with adults, have been shown in previous analysis because children have higher VL and require longer times to achieve an uVL [17]. Furthermore, our study recruit a high number of children older than 3 years, 74%, who have been taken ART for long periods of time and whose rates of virologic failure were higher that those reported in infant under 3 years, (77% vs. 56%). Besides, regarding to the unpredictability plasma concentrations of NFV that have been reported [6], the lack of therapeutic drug monitoring (TDM) measurement and analysis in a normal cohort (not in clinical trial) could have reduced the effectiveness in our study.

During the three years, 45% change the line of HAART and 38% interrupt NFV. These patients have similar characteristics at baseline so the use of a resistance test may have allowed selecting the most appropriate NRTI for each patient. Moreover, at baseline only 1/3 of children included 2 new NRTI in HAART line and 1/3 did not take any new NRTI. High virologic failure at the end of our study is probably due to previous resistance mutations to NRTI. Besides, new NNRTI or PI were added as second HAART line and some patients changed NRTI during the follow-up. However, the global tendency of VL was not improved which could be explained by the appearance of new resistance mutations to NRTI or cross resistance to PI. It is probable that if a resistance assay would have been used to guide changes of ART, virological response would have been higher.

Our study reveals that NFV is safe because only four patients experienced side effects. These results are considerably minor than other studies that reveal gastrointestinal side effects related to the use of NFV in 18% patients [18]. On each follow-up visit only 40% achieve uVL. However, 90% have a decreased in VL >1 log_10 _and 70% have an uVL during the three years follow-up. Only VL at baseline was an important factor to predict VL control during the follow-up, as others studies have also shown [8].

## Conclusion

In conclusion, when selecting an antiretroviral combination we need to consider short and long toxicities, as well as the immunologic, virologic and clinical benefit that a patient can achieve. Our results show that NFV is an effective and well tolerate drug for HIV infection among children. However, more studies which include measurements as TDM or resistance test are needed to evaluate the use of this drug in paediatric population.

## Competing interests

**Potential conflicts of interest: **The authors declare that they have no competing interests.

## Authors' contributions

SR had primary responsibility for protocol development, patient screening, enrolment, outcome assessment, preliminary data analysis, and contributed to the writing of the manuscript. BL contributed to the writing of the manuscript. JM Bellón participated in analytic framework for the study, and contributed to the writing of the manuscript. RR had primary responsibility for the collecting and recording data, and contributed to the writing of the manuscript. Paediatricians: MIJ, MDG, JL, JTR and MJM were responsible for patient screening, and contributed to the writing of the manuscript. MAMF supervised the design and execution of the study, the final data analyses, and the writing of the manuscript. All authors read and approved the final manuscript.

## Pre-publication history

The pre-publication history for this paper can be accessed here:


